# Explainable machine learning for predicting hospital employees' quality of life using psychosocial work environment data

**DOI:** 10.3389/fpubh.2025.1529802

**Published:** 2025-12-01

**Authors:** Arwa Alumran, Bashayer Alshahrani, Nida Aslam, Irfan Ullah Khan, Rana AlShedayed, Dina AlFrayan, Rand AlEssa, Samiha Mirza, Fatima Alshakhs

**Affiliations:** 1 Health Information Management and Technology, College of Public Health, Imam Abdulrahman bin Faisal University, Dammam, Saudi Arabia; 2 Radiology Department, John Hopkins Aramco Hospital, Khobar, Saudi Arabia; 3 Department of Computer Science, College of Computer Science and Information Technology, Imam Abdulrahman bin Faisal University, Dammam, Saudi Arabia

**Keywords:** machine learning, quality of life, prediction, psychosocial work environment, Explainable Artificial Intelligence

## Abstract

Health-Related Quality of Life (HRQL) embodies the impact of an individual's health on their ability to live a fulfilling life. Quality of Life (QoL) is influenced by a range of factors, including physical functioning and wellbeing, psychological functioning, work environment (WE), lifestyle, and social relations. Various studies have found that job-related factors can be an essential predictor of an individual's HRQL. Furthermore, the Psychosocial Work Environment (PWE) can affect workers' wellbeing and contribute to the company's sustainability. PWE and QoL influence the quality of health services provided by healthcare providers. Therefore, the relationships among QoL, PWE, and healthcare quality need to be assessed to identify factors that improve overall patient healthcare service quality. This relationship has not been extensively evaluated in the Saudi context. Therefore, in the current study, we aimed to employ machine learning (ML) techniques to predict employee QoL using PWE data from a hospital in the Kingdom of Saudi Arabia (KSA). Several ML models have been developed to predict HRQL effectively and their significant attributes; the experiments were carried out with and without feature engineering. The Naïve Bayes (NB) classifier achieved the highest precision of 1.0 (95% CI: 0.81–1.0) in predicting employees' QoL using PWE and demographic variables. The selected Work Environment (WE) features, identified using the Xverse voting selector with the SVM classifier achieved the best results, with accuracy, recall, precision, F1, and receiver operating characteristic (ROC) reaching 0.92 (95% CI: 0.88–0.95), 0.90 (95% CI: 0.86–0.98), 0.95 (95% CI: 0.86–0.99), 0.92 (95% CI: 0.88–0.95), and 0.9, respectively. *Post-hoc* Explainable Artificial Intelligence (XAI) was used to alleviate the black-box nature of SVM and add transparency to the model. In conclusion, this study provides a robust, explainable tool for predicting employee QoL that can help healthcare organizations improve quality.

## 1 Introduction

Health-Related Quality of Life (HRQL) is multidimensional and influenced by factors such as physical and psychological health, work environment, lifestyle, and social relations (1). HRQL, as per the World Health Organization (WHO) (2), is the culture and values of an individual's perception and its relationship with standards, goals, and concerns. Several factors have been discussed in previous studies that can affect HRQL, such as work attitude, job satisfaction, organizational commitment, and job content; hence, these factors can be used as predictors for an individual's HRQL (3). Several studies have been conducted related to the nurses' job satisfaction, yet it is relatively limited and needs further investigation (4).

Furthermore, in the literature, it is reported that the work environment has a high impact on the employee's productivity (5). Collaborative workplace environments have a positive impact on employee wellbeing, whereas toxic environments lead to adverse outcomes. They can increase organizational costs by reducing employee productivity (6–8). Furthermore, group dynamics within the organization also play a vital role in employees' quality of life (QoL) and workplace comfort (9). Assessing the quality of social communication is an essential aspect of enhancing the overall psychosocial work environment, especially in the health sector. Many organizations focus solely on profit, based on the cost and quality of the job performed or the product produced, but the central driving factor for the profit of any organization is its employees, who are usually ignored and treated as performers (5). To create a healthy work environment, it should be a collaborative effort among employers, employees, and even society's health policymakers.

Research indicated that psychological and behavioral factors have a substantial impact on both the wellbeing of healthcare professionals and the safety of patients (6, 7, 10). An unhealthy psychosocial environment will pose a risk to the organization's reputation and the employee's outcome. Behavioral modification and physical activity have a direct impact on QoL, improving an individual's HRQL (8), and are a basis to calculate quality-adjusted life-years (QALYs) (11). Moreover, cognitive demands are the degree to which the job requires employees to be highly concentrated on their work, which requires organizational support and commitment (12). The relationship between an employee and a leader has a potential impact on the employee's outcomes in an academic institute (13, 14). In the Information Communication and Technology (ICT) era, minimal contact between employees and the community is the norm (15). According to Sievers (16), the community can create motivation. Furthermore, the most frequent work stressor is the behavioral interaction between staff (9). Supervisor interactions were shown to be associated with emotional exhaustion or burnout of the employees (17). Motivation is defined by (18) as “psychological processes that cause the arousal, direction, and persistence of voluntary actions that are goal-directed” (19). The best performance is achieved by an organization and its committed employees through motivation. Employees usually prefer incentives and rewards as motivational factors, while the terms recognition and reward are typically used simultaneously (20). The job insecurity leads to a negative impact on health and behavior, such as a low level of innovation and creativity, less employment stability, and fewer initiatives, because they cannot afford the risk (21, 22). Though job security and freedom are challenging factors in the workplace, if these two factors are ensured for employees in any organization, this will result in increased productivity (23). Job security is one of the critical aspects of the Psychosocial Work Environment (PWE) that needs to be assessed in relation to the person's HRQL. A positive PWE factor in any organization strongly contributes to job satisfaction. According to Gibbs et al. (24), more productive employees receive more formal training, are more satisfied, and are less likely to have conflicts with others and their supervisors. According to the American Psychological Association, money and work environment are the top two factors that cause stress to employees (25). The work environment can cause negative employee attitudes and reduced performance and outcome of care (25, 26). The WHO healthy workplace model identifies five key points to create a healthy work environment: leadership commitment and engagement; involve workers and their representatives; business ethics and legality; use a systematic and comprehensive process to ensure effectiveness and continual improvement; and sustainability and integration (2). Besides, the European Agency for Safety and Health at Work identifies the need to improve the physical work environment (27). According to Rezagholi (28), PWE can affect workers' wellbeing and the company's future sustainability. Interest and investment in employees' psychological health call for training and evaluation within and outside the organization. Moreover, Abdi et al. (7) found that job satisfaction and demand had a strong association with performance. Similarly, the analysis of psychosocial factors conducted on the cardiac care unit professionals found that to improve the organization's outcome, one of the significant factors is to provide incentives and encouragement to the employees (29). Similar findings among Iranian nurses aimed at predicting resilience: they found that some psychosocial factors, such as transparency, good leadership, encouragement, and respect, have the potential to enhance nurses' outcomes (30). Mendoza Bernal et al. (31) also predicted nurses' resilience after the first and second waves of COVID-19 in Spain. Different levels of resilience have been observed across all variables except anxiety. Ali et al. (32) found an inverse correlation between workplace ostracism and nurses' work behavior using the dataset collected from the Saudi Arabia (KSA) hospital. A multiple linear regression model was used to predict nurses' QoL. Recently, the authors have explored the association between the WE and the decision-making of clinical nurses. The study found a strong correlation between PE and clinical decision-making (33). Quality is a sensitive and dynamic domain in healthcare, and the PWE is one of the most influential factors that could directly affect the quality of services provided. Measuring the effect of work-related qualitative and cognitive demands on employee productivity is relatively less explored in the Kingdom of Saudi Arabia (KSA) context, particularly in the health sector. Therefore, in the current study, we attempt to identify and predict the hospital employees' QoL using the PWE. Understanding the PWE within the KSA healthcare system is significant for opening new avenues for upcoming workplace interventions and elevating quality healthcare practices. Moreover, KSA's Vision 2030 highlights strategies focused on the national revolution and on enhancing QoL in KSA, which lies at the heart of the three main pillars of Vision 2030: “A Vibrant Society: focused on preserving culture, promoting entertainment and sports, and enhancing quality of life.” This pillar further encompasses improving healthcare services, promoting preventive care, advancing hospital and digital health systems, and increasing life expectancy. Hence, this study supports a key objective of Vision 2030 by contributing to the understanding and improvement of QoL. The QoL for healthcare providers and the PWE directly affect the quality of health services provided. Therefore, in this study, we will assess the relationship between QoL and PWE to identify areas for improvement in PWE. Enhancing the employee's PWE, their QoL scores will improve, which in turn will improve the overall healthcare service quality provided to patients. The study proposes a robust, transparent machine learning (ML) model using *post-hoc* Explainable Artificial Intelligence (XAI) to predict employee QoL, thereby helping healthcare organizations improve quality. XAI adds transparency to the black-box ML models (34). To the author's knowledge, XAI has not been previously investigated for predicting HRQL.

## 2 Materials and methods

**Figure 1:**
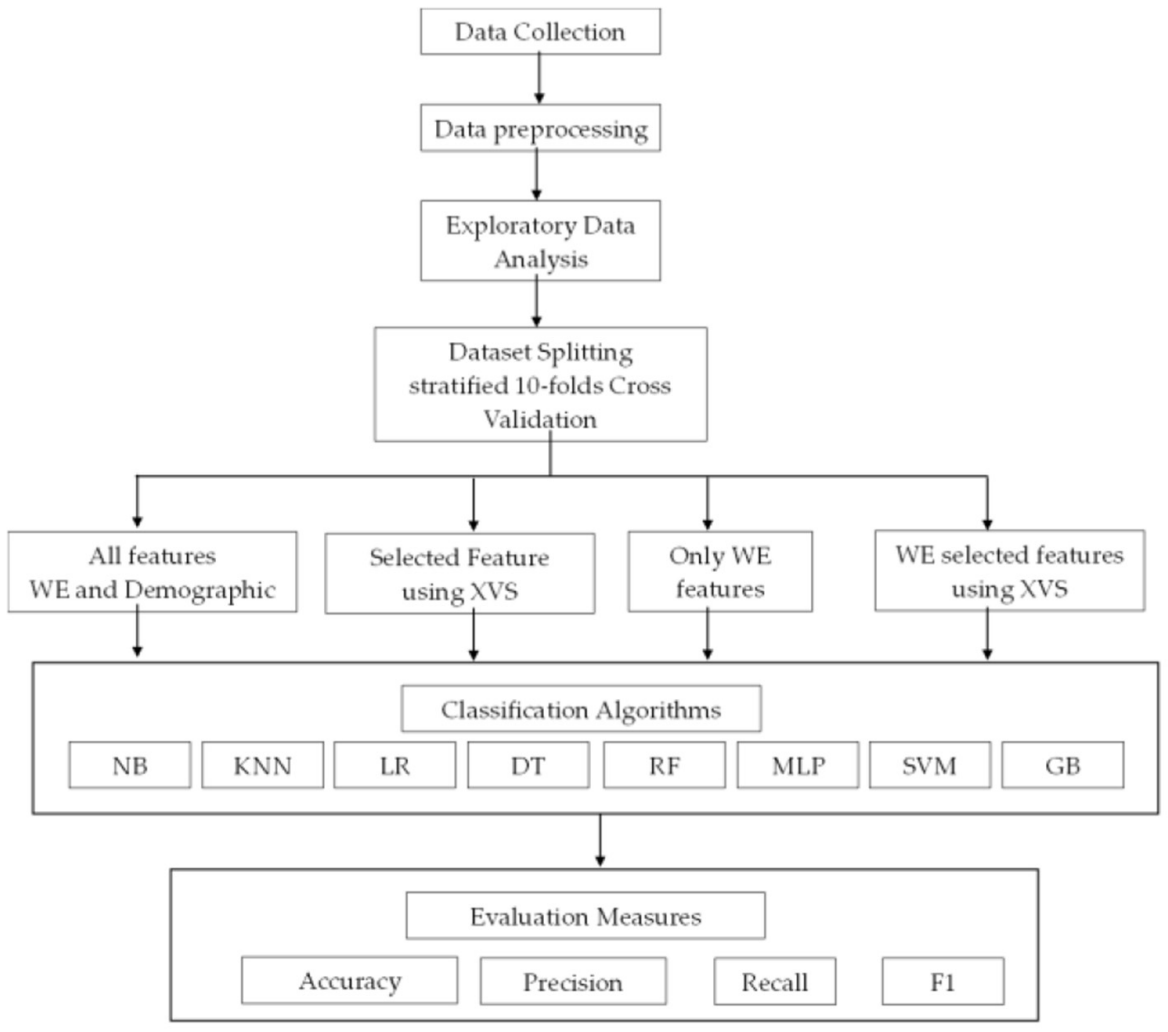
Proposed methodology framework.

This section presents a description of the proposed methodology, including dataset collection, data processing, exploratory analysis of the dataset, prediction models, and evaluation measures. Figure 1 presents the proposed methodology.

### 2.1 Dataset collection

This is a quantitative cross-sectional study design using an online survey. The target population is all healthcare providers in King Fahd University Hospital (KFUH), Eastern Province, KSA. The dataset was collected from physicians, nurses, administrative personnel, and allied health professionals (radiology, pharmacy, laboratory, physical therapy, nutrition, and respiratory therapy). Participants' HRQL scores were collected along with their PWE scores and some personal information (e.g., gender, years of experience).

The RAND 20-item scale was used to measure the HRQL score (35). The PWE scale was adapted from (36, 37). In both scales, higher scores indicate better HRQL and better PWE. Ethical approval was obtained from the Institutional Review Board at Imam Abdulrahman bin Faisal University (IAU; IRB Number: PGS-2019-03-035). The survey was conducted confidentially, with no personal information disclosed to ensure participants' privacy.

### 2.2 Exploratory data analysis

The dataset contains 27 attributes across two categories: personal information and PWE. Table 1 presents the information gain for the most predictable features. The dataset includes 128 samples, with 63 in the bad category and 65 in the good category. The dataset does not contain any missing data. Due to the limited variation in the data values, no standardization or normalization techniques have been applied. During preprocessing, all categorical attributes were encoded into numerical values using the LabelEncoder utility from the scikit-learn library. Most of the features in Table 1 are related to the work environment (WE); however, only two attributes relate to personal information, namely profession and qualification. The highest information gain attribute is profession. HRQL is dependent on the employee's rank.

**Table 1:** Highest predictability features.

**Attributes**	**Values**	**Info. gain**
Profession	Administrative	0.208
	Physician	
	Nursing	
	Allied Health^*^	
Contradictory demands placed on you at work: (Role conflicts)	Very large extent	0.101
	Large extent	
	Somewhat	
	Small extent	
Your highest educational qualification	Primary school	0.093
	Secondary or high school	
	Bachelor's degree	
	Master's degree	
	PhD degree or higher (Or Board-certified)	
You work in isolation from your colleagues: (Social community at work)	Always	0.078
	Often	
	Some of the time	
	Rare	
	Never	
You have the possibility of learning new things through your work: (Possibilities for development)	Very large extent	0.053
	Large extent	
	Somewhat	
	Small extent	
	Very small extent	
Your superior talks with you about how you will carry out your work: (Role conflicts)	Always	0.049
	Often	
	Some of the time	
	Rare	
	Never	
Are you worried about becoming unemployed: (Job insecurity)	Very large extent	0.045
	Large extent	
	Somewhat	
	Small extent	
	Very small extent	
Feedback regarding your work: (Recognition)	Very large extent	0.034
	Large extent	
	Somewhat	
	Small extent	
	Very small extent	

^*^Allied health (pharmacy, laboratory, radiology, physical therapy, respiratory therapy, nutrition).

### 2.3 Classification model

The current study aims to develop an intelligent ML-based model to predict the hospital employees' QoL. Naïve Bayes (NB), K Nearest Neighbor (KNN), Logistic Regression (LR), Decision Tree (DT), Random Forest (RF), Multi-Layer Perceptron (MLP), Gradient Boosting (GB), and Support Vector Machine (SVM) were used in the study as the prediction models. A grid search mechanism was used to find the optimal parameters. A description of the deployed models is presented in the section below.

#### 2.3.1 Decision tree

DT is a decision-support technique used for developing classification and regression models. It has a tree-like structure in which every internal node represents a test on an attribute, the branches represent the outcomes of those tests, the terminal/leaf node represents a class label, and the top decision node is the root node of the tree (38). Each path taken from the root to the leaf represents a sequence of data splits that lead to a Boolean outcome. Having a high splitting power at each stage of the tree creates the shortest possible tree. The algorithm computes the entropy of the data set, using the formula below:


$$\begin{array}{l} H\left(Set\right)=\;-\;P_{1}\;\times log_{2}\;P_{1}-\;P_{2}\;\times log_{2}\;P_{2} \end{array}$$ (1)

Where *P*_1_ is the proportion of the first decision and so on.

To measure the splitting power for each attribute, the information gain formula can be written as:


$$\begin{array}{l} Gain(A)=H(Set)-(w_{1}\times H(a_{1})+w_{2}\\ \;\times H(a_{2})+\ldots +w_{m}\times H(a_{m})) \end{array}$$ (2)

**Figure 2:**
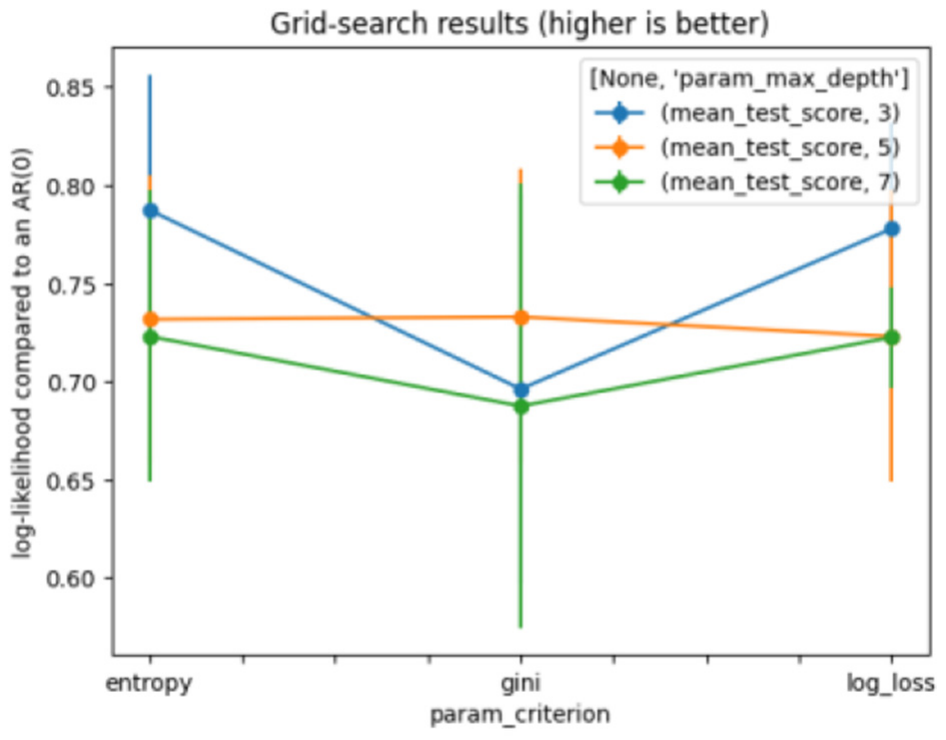
Grid search for decision tree.

Where *a*_1_, *a*_2_ … *a*_*m*_ represent the many values of attribute A, and *w*_1_, *w*_2_ … *w*_*m*_ represents the weights of the subset splits using the same values of attribute A (38). GridSearchCV was applied to find the optimal parameter. The model achieves good, stable results with the settings shown in Table 2. Figure 2 shows the results of the DT grid search.

**Table 2:** Optimal parameters for decision tree.

**Parameters**	**Value**
Criterion	gini
Splitter	best
max_depth	5

#### 2.3.2 Random forest

The Random Forest (RF) algorithms form a family of decision trees that operate by aggregating the classification results from multiple decision trees. RF uses binary splits frequently, which split the tree into homogeneous or near-homogeneous nodes. The continuous splitting of the parent node improves the homogeneity of the child nodes. Generally, RF trees are grown deeply; therefore, the number of trees often reaches thousands (38). The growing moves in two stages and uses a random subset of variables at each node to find the best split.

The RF method is built through the following steps:

1. First, it extracts several sample trees from the raw data.
2. For each sample, a regression tree is grown, then for each node, a random subset of variables is chosen to predict the best split.
3. Finally, predictions of all tresses are gathered, and the classification with the most votes is chosen to predict the new data.

**Figure 3:**
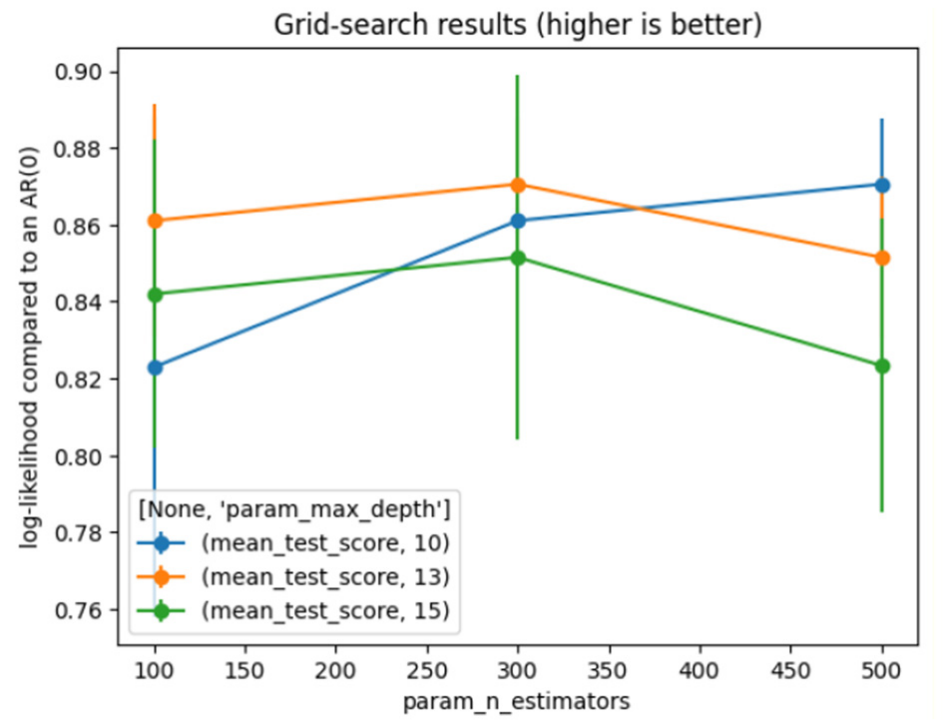
Grid search for random forest.

The model achieves good and stable results with the settings shown in Table 3. Figure 3 represents the results of the grid search on RF.

**Table 3:** Optimal parameters for random forest.

**Parameters**	**Value**
n_estimators	300
max_depth	13
min_samples_split	5

#### 2.3.3 Logistic regression

Logistic Regression (LR) is an ML algorithm and statistical model that utilizes the maximum-likelihood ratio approach for classification and regression (38). It is based on probability theory and predictive analysis algorithms. The advantage of LR is that it employs a straightforward probabilistic classification formula. Conversely, the disadvantage of the linear regression model is that it is ineffective for non-linear problems. LR can be expressed as follows:


$$\begin{array}{l} \log\left[\frac{y}{1-y}\right]=\;b_{0}+b_{1}x_{1}+b_{2}x_{2}+b_{3}x_{3}+\;\ldots +b_{n}x_{n} \end{array}$$ (3)

**Figure 4:**
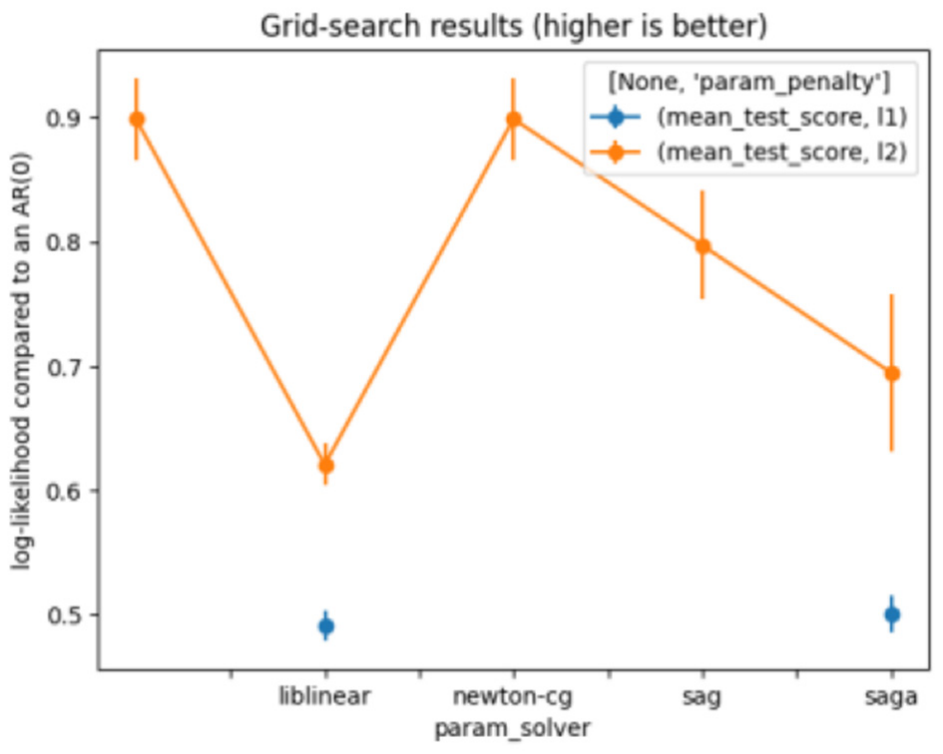
Grid search for logistic regression.

The LR parameters used are shown in Table 4. Figure 4 presents the results of the grid search on LR.

**Table 4:** Optimal parameters for logistic regression.

**Parameters**	**Value**
Penalty	12
max_iter	10,000
Solver	“newton-cg”

#### 2.3.4 Naïve Bayes

A Naive Bayes (NB) classifier is a simple probabilistic ML algorithm used for task classification. It has performed effectively across a range of complex real-world applications, including sentiment analysis, real-time prediction, spam filtering, and medical diagnosis. Bayesian classification methods are used to build the NB. Nevertheless, due to the unrealistic expectations that all predictors are independent and essential, the performance of this classifier may be hindered in some circumstances (38).

Bayes' Theorem is represented by the equation below:


$$\begin{array}{l} P\left(A|B\right)=\frac{P\left(B|A\right)P\left(A\right)}{P\left(B\right)}\; \end{array}$$ (4)

#### 2.3.5 K nearest neighbors

K Nearest Neighbors (KNN) is a fundamental supervised learning algorithm used in ML. KNN is versatile, considering it can be used for classification and regression problems. Primarily, this algorithm works by classifying incoming data points based on the similarity of previously stored data points (neighbors). To calculate the nearest neighbor, distance measures such as the Euclidean, Manhattan, and Minkowski distances are used; see the equations below.

Euclidean distance:


$$\begin{array}{l} D\left(x,y\right)=\sqrt{\sum_{i=1}^{n}{\left(x_{i}-y_{i}\right)}^{2}} \end{array}$$ (5)

Manhattan distance:


$$\begin{array}{l} D\left(x,y\right)=\sum_{i=1}^{k}|x_{i}-y_{i}|\; \end{array}$$ (6)

Minkowski distance:


$$\begin{array}{l} D\left(x,y\right)={\left({\sum_{i=1}^{n}|x_{i}-y_{i}|}^{p}\right)}^{\frac{1}{p}} \end{array}$$ (7)

KNN is best employed when the data volume is low, since the algorithm's computational cost increases with volume.

#### 2.3.6 Multi-layer perceptron

**Figure 5:**
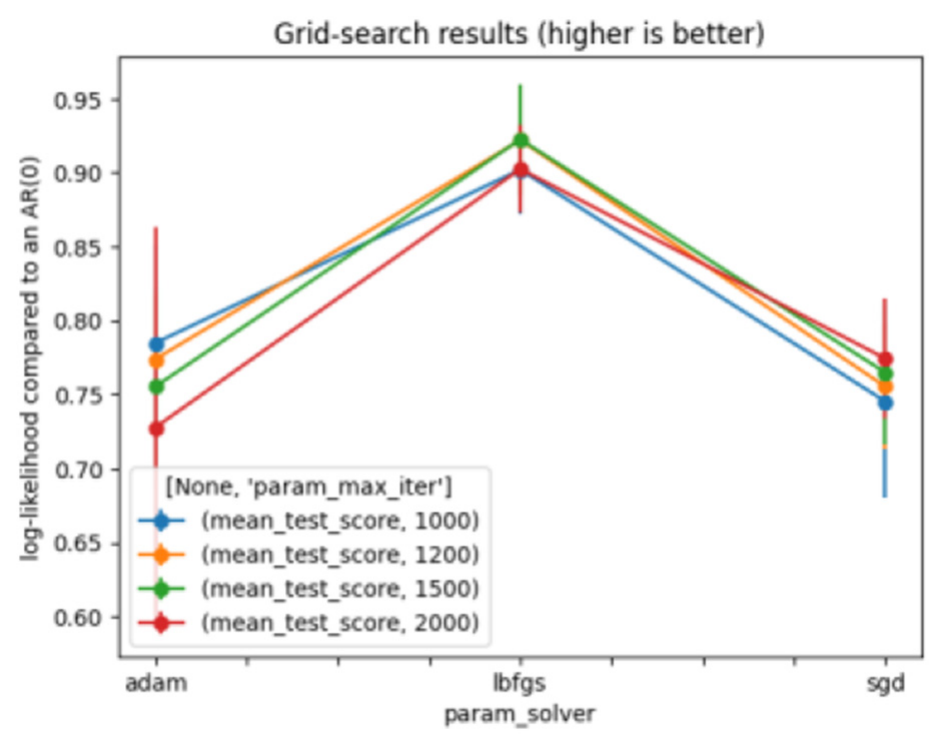
Grid search for multi-layer perceptron.

A Multi-Layer Perceptron (MLP) is a deep learning technique that uses a feedforward artificial neural network (ANN) to produce a set of outputs from a set of inputs. Several layers of input nodes are connected, forming a directed graph between the input and output layers of an MLP, indicating that the signals travel only in a unidirectional way across the nodes. MLP uses the backpropagation algorithm for supervised learning to train the network (38). Table 5 depicts the optimal parameters for MLP. Figure 5 shows the results of the grid search on MLP.

**Table 5:** Optimal parameters for multi-layer perceptron.

**Parameters**	**Value**
Solver	lbfgs
max_iter	1,200
hidden_layer_sizes	13

#### 2.3.7 Support vector machine

**Figure 6:**
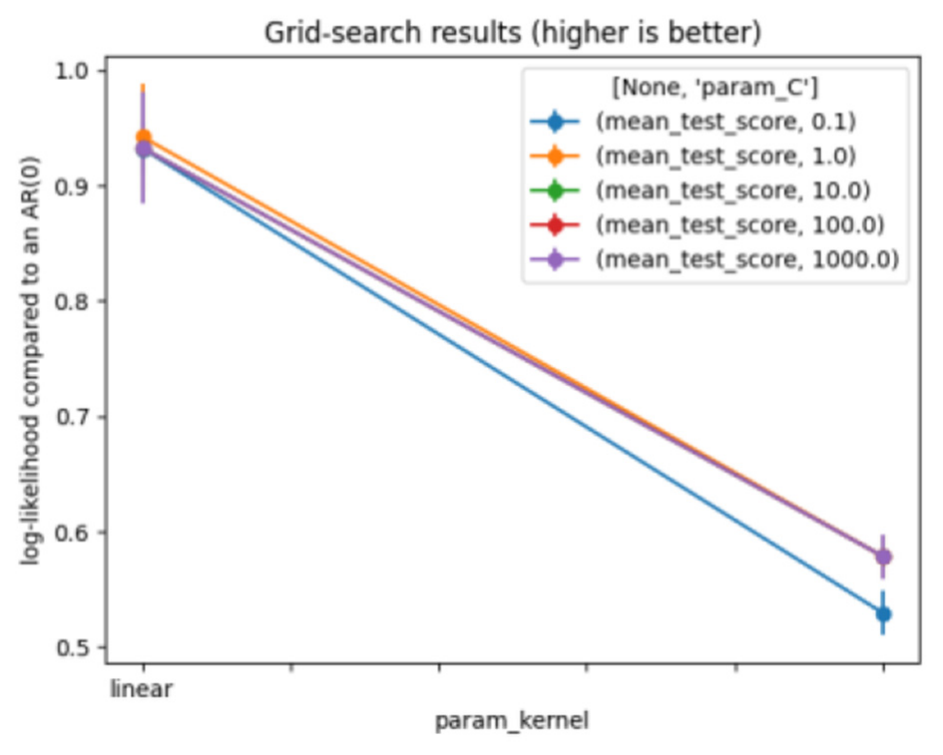
Grid search for support vector machine.

A Support Vector Machine (SVM) is a supervised learning model used for regression (SVR), classification (SVC), data analysis, and outlier detection. It can also be used for unsupervised learning, where it is known as support vector clustering. The SVM settings generate an n-dimensional vector space, where each dimension (x, y, z, etc.) corresponds to a feature of each point, aiming to train a model to classify new out-of-sample data. SVM algorithms use various types of linear or non-linear kernel functions [polynomial, Radial Basis Function (RBF), and sigmoid] to offer a bridge from non-linearity to linearity (38). The model produces satisfactory and stable results with the following settings, as shown in Table 6. Figure 6 represents the results of the grid search on SVM.

**Table 6:** Optimal parameters for support vector machine.

**Parameters**	**Value**
Kernel	Linear
C	10

#### 2.3.8 Gradient boosting

**Figure 7:**
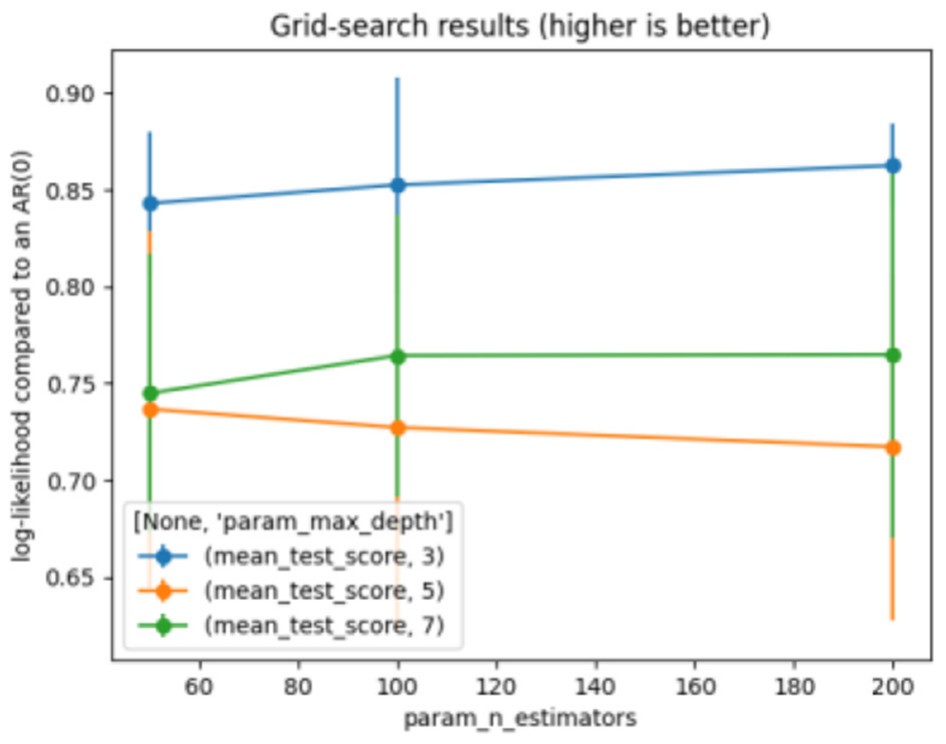
Grid search for gradient boosting.

A Gradient Boosting (GB) is an ensemble classification technique in ML. It is a specific type of algorithm that classifies the task given to a machine. The concept of GB comes from taking a weak hypothesis or weak learning algorithm and turning it into a series of trials that will increase the learner's power. The method employed is as follows: the set of incorrectly classified data is used to generate a new weak learner, which is then checked; only the correctly classified examples are retained. They are defined by adjusting the data and model weight. GB classifiers aim to reduce the loss, or the gap, between the training example's actual class value and the class value predicted. The GB classifier is therefore based on a loss function and supports uniform loss functions, provided they are differentiable. The parameter values for the proposed GB are shown in Table 7. Figure 7 represents the results of the grid search on GB.

**Table 7:** Optimal parameters for gradient boosting.

**Parameters**	**Value**
learning_rate	0.05
max_depth	3

### 2.4 Evaluation measures

The standard metrics used to evaluate the performance of the models were accuracy, F1-Score, precision, and recall (sensitivity) (39). A 95% Confidence Interval (CI) for each metric was estimated using the bootstrap method with 1,000 resamples. This technique provides a robust measure of uncertainty.

#### 2.4.1 Accuracy

Accuracy is an evaluation metric that measures the total number of correct predictions to determine how accurate is the model. Accuracy can be expressed as follows:


$$\begin{array}{l} Accuracy=\frac{TP+TN}{TP+FN+TN+FP} \end{array}$$ (8)

#### 2.4.2 Recall

Recall is the proportion of the total relevant results that the model accurately classifies. It is essentially a measure of a model's ability to identify True Positives correctly. Recall can be expressed as follows:


$$\begin{array}{l} Recall=\frac{TP}{TP+FN} \end{array}$$ (9)

#### 2.4.3 Precision

Precision, also known as Positive Predictive Value (PPV), is a measure of how many accurate predictions have been made. Precision can be expressed as follows:


$$\begin{array}{l} PPV=\frac{TP}{TP+FP}\; \end{array}$$ (10)

#### 2.4.4 F1-Score

The F1-Score is described as the harmonic mean of precision and recall, which can be mathematically expressed as follows:


$$\begin{array}{l} F1-Score=\frac{2\;*\left(Recall\;*\;Precision\right)}{Recall+Precision} \end{array}$$ (11)

#### 2.4.5 Receiver operating characteristic (ROC)

The ROC curve plots the true positive rate against the false positive rate at different thresholds. It is the most used graphical plot for binary classification.

## 3 Experiments and results

The experiments were conducted on Windows 10, 64-bit architecture, using the Jupyter notebook. The models were developed using the Python programming language (ver. 3.12.4). Several Python libraries were used, such as Numpy (ver. 1.26.4), Matplotlib (ver. 3.7.5), Sklearn (ver. 1.4.2), Pandas (ver. 2.1.4), lime (ver. 0.2.0.1), and Dalex (ver. 1.7.2). The data contains 27 features (i.e., gender, profession, age, education, shift, experience, nationality, and 20 variables related to PWE), QoL is the targeted variable, and it is discrete with binary values. Stratified 10-fold Cross Validation (CV) for partitioning the data for training and testing. The dataset was divided into 10 folds, and stratified sampling was used. The process was repeated 10 times, with each iteration using onefold for testing and the remaining folds for training. The results were calculated for each iteration. The result was computed as the average across folds. This method reduces the chance of model overfitting and provides more reliable results. Classification models were developed and evaluated using the following standard measures: accuracy, F1 score, precision, and recall (sensitivity). However, for the best-performing experiment 4, the ROC curve has been included. Several sets of experiments were performed with all the features, a selected category of features, and features selected using Xverse VotingSelector. Xverse is an abbreviation for X-Universe (40). This technique uses a group of transformers for feature selection. The Xverse voting selector uses the voting approach to select the most pertinent features. The primary advantage of this approach is that it relies on multiple feature selection algorithms rather than a single one, thereby reducing the risk of model overfitting. Some feature selection algorithms include recursive feature elimination, chi-square, Random Forest, weight of evidence, ANOVA, and correlation matrix. The results achieved and selected features for each experiment are discussed in the sections below.

**Figure 8:**
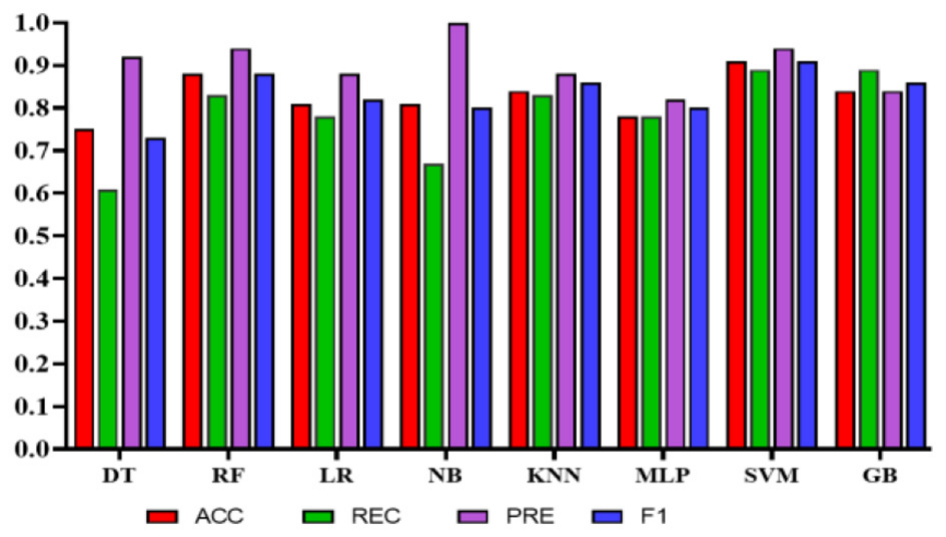
Comparison of the proposed models using demographic and work environment features.

Experiment 1: In the first experiment, all features were used without feature selection. Table 8 presents the results of experiment 1. The comparison among the classifiers is shown in Figure 8. The total number of features used in this experiment was 27. The NB classifier achieved the highest precision of 1.0 (95% CI: 0.81–0.92) to predict employees' QoL using variables from the PWE and demographics. However, recall value achieved using NB was 0.67 (95% CI: 0.58–0.96). In comparison, SVM achieved the highest accuracy, recall, and F1 score of 0.91 (95% CI: 0.87–0.97), 0.89 (95% CI: 0.83–0.97), 0.91 (95% CI: 0.86–0.97), respectively, with the precision of 0.94 (95% CI: 0.79–0.95).

**Table 8:** Models' performance using WE and demographic features (without feature engineering).

**Classifier**	**Accuracy (95% CI)**	**Recall (95% CI)**	**Precision (95% CI)**	**F1 (95% CI)**
**Without feature engineering (WE** + **demographic features)**				
DT	0.75 (0.72–0.87)	0.61 (0.56–0.93)	0.92 (0.83–0.99)	0.73 (0.66–0.88)
RF	0.88 (0.82, 0.95)	0.83 (0.82, 1.00)	0.94 (0.80–0.97)	0.88 (0.81–0.96)
LR	0.81 (0.70–0.89)	0.78 (0.65–0.91)	0.88 (0.76–0.92)	0.82 (0.69–0.92)
NB	0.81 (0.77–0.96)	0.67 (0.58–0.96)	1 (0.81–1.00)	0.80 (0.78–0.96)
KNN	0.84 (0.78–0.88)	0.83 (0.80–0.97)	0.88 (0.78–0.92)	0.86 (0.80–0.89)
MLP	0.78 (0.69–0.88)	0.78 (0.74–0.93)	0.82 (0.73–0.89)	0.80 (0.71–0.88)
SVM	0.91 (0.87–0.97)	0.89 (0.83–0.97)	0.94 (0.79–0.95)	0.91 (0.86–0.97)
GB	0.84 (0.78–0.91)	0.89 (0.79–0.99)	0.84 (0.77–0.92)	0.86 (0.79–0.92)

**Figure 9:**
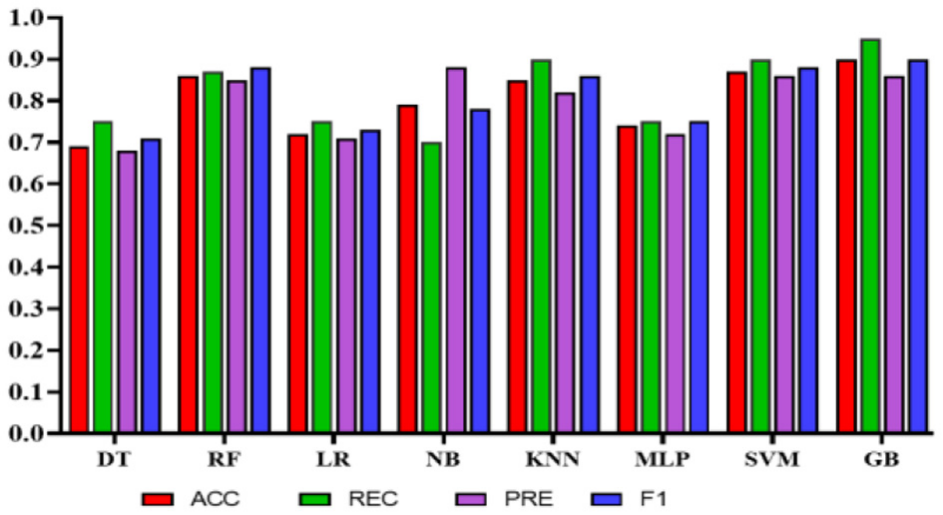
Comparison of the proposed models with selected features using Xverse voting selector.

Experiment 2*:* To reduce the number of features further while preserving the model's high accuracy, feature engineering was applied using Xverse Voting Selector on WE and demographic features. The total number of features used in this experiment was 18. Three demographic features (gender, age, and experience) were selected during feature selection, along with 15 work environment features: Q1, Q3, Q4, Q5, Q6, Q7, Q8, Q10, Q11, Q13, Q14, Q15, Q16, Q18, and Q19. The questionnaire details are provided in the Supplementary File S1. With reduced features, the NB's performance degrades in terms of precision, accuracy, and F1 score; however, recall improves. While in this experiment GB achieved the best values in all the metrics with an accuracy of 0.90 (95% CI: 0.82–0.92), a recall of 0.95 (95% CI: 0.88–0.99), a precision of 0.86 (95% CI: 0.76–0.95), and an F1 score of 0.90 (95% CI: 0.84–0.93). The results are depicted in Table 9. Furthermore, the comparison is represented in Figure 9.

**Table 9:** Model's performance using WE and demographic features (using Xverse voting selector).

**Classifier**	**Accuracy (95% CI)**	**Recall (95% CI)**	**Precision (95% CI)**	**F1 (95% CI)**
**With feature engineering (WE** + **demographic features)**				
DT	0.69 (0.63–0.85)	0.75 (0.70–0.94)	0.68 (0.65–0.88)	0.71 (0.79–0.86)
RF	0.86 (0.82–0.92)	0.87 (0.83–0.98)	0.85 (0.77–0.95)	0.88 (0.84–0.93)
LR	0.72 (0.67–0.86)	0.75 (0.65–0.84)	0.71 (0.64–0.76)	0.73 (0.62–0.83)
NB	0.79 (0.77–0.94)	0.70 (0.68–0.96)	0.88 (0.77–1.0)	0.78 (0.71–0.93)
KNN	0.85 (0.82–0.93)	0.90 (0.88–0.97)	0.82 (0.64–0.98)	0.86 (0.83–0.93)
MLP	0.74 (0.68–0.82)	0.75 (0.71–0.90)	0.72 (0.64–0.82)	0.75 (0.72–0.84)
SVM	0.87 (0.83–0.99)	0.90 (0.86–0.97)	0.86 (0.81–0.99)	0.88 (0.84–0.99)
GB	0.90 (0.82–0.92)	0.95 (0.88–0.99)	0.86 (0.76–0.95)	0.90 (0.84–0.93)

**Figure 10:**
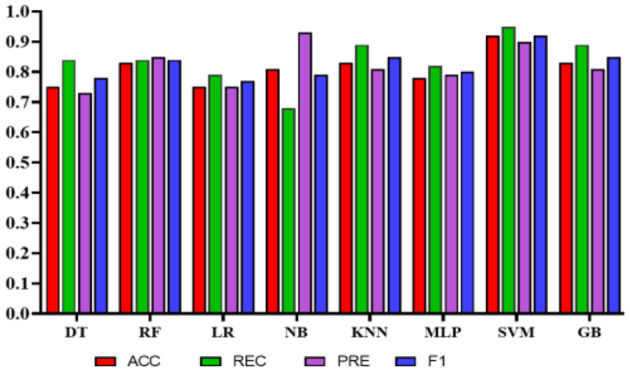
Comparison of the proposed models using only work environment features.

Experiment 3: In the third experiment, WE features were investigated separately to assess the classifiers' performance in predicting QoL. Table 10 shows the performance of the classifiers using WE features only without applying any feature engineering. The total number of features used in this experiment was 20. In this case, NB achieved the highest precision of 0.93 (95% CI: 0.77–0.99). However, SVM obtained the highest accuracy, recall, and F1 scores of 0.92 (95% CI: 0.89–0.99), 0.95 (95% CI: 0.92–1.0), and 0.92 (95% CI: 0.89–0.99), respectively. Figure 10 represents the comparison of different classifiers in experiment 3.

**Table 10:** Model's performance using WE features (without feature engineering).

**Classifier**	**Accuracy (95% CI)**	**Recall (95% CI)**	**Precision (95% CI)**	**F1 (95% CI)**
**Without feature engineering (work environment features only)**				
DT	0.75 (0.72–0.86)	0.84 (0.74–0.94)	0.73 (0.71–0.90)	0.78 (0.71–0.87)
RF	0.83 (0.82–0.92)	0.84 (0.81–0.98)	0.85 (0.77–0.95)	0.84 (0.81–0.93)
LR	0.75 (0.60–0.86)	0.79 (0.68–0.92)	0.75 (0.55–0.94)	0.77 (0.69–0.89)
NB	0.81 (0.77–0.94)	0.68 (0.57–0.96)	0.93 (0.77–0.99)	0.79 (0.75–0.93)
KNN	0.83 (0.81–0.93)	0.89 (0.81–0.94)	0.81 (0.77–0.98)	0.85 (0.83–0.93)
MLP	0.78 (0.72–0.85)	0.82 (0.74–0.88)	0.79 (0.67–0.88)	0.80 (0.69–0.87)
SVM	0.92 (0.89–0.99)	0.95 (0.92–1.0)	0.90 (0.87–0.99)	0.92 (0.89–0.99)
GB	0.83 (0.81–0.92)	0.89 (0.76–0.95)	0.81 (0.76–0.95)	0.85 (0.82–0.93)

**Figure 11:**
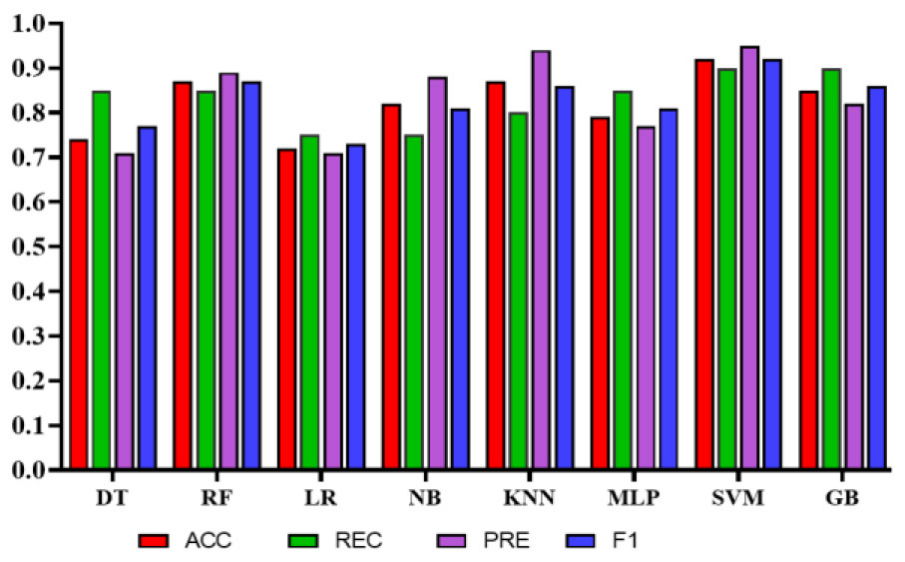
Comparison of the proposed models using Xverse voting selector among work environment features.

**Figure 12:**
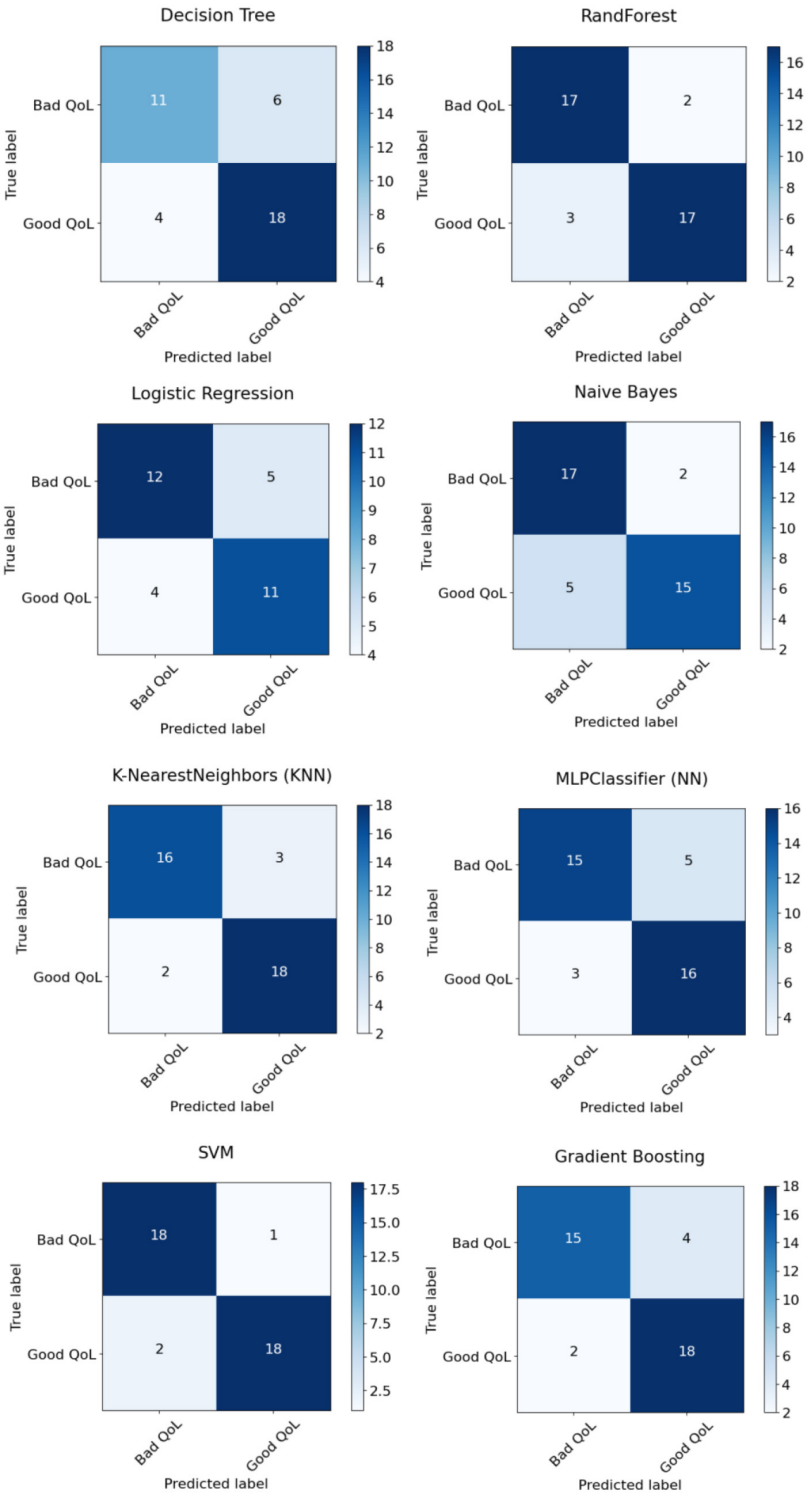
Experiment 4 confusion matrix.

**Figure 13:**
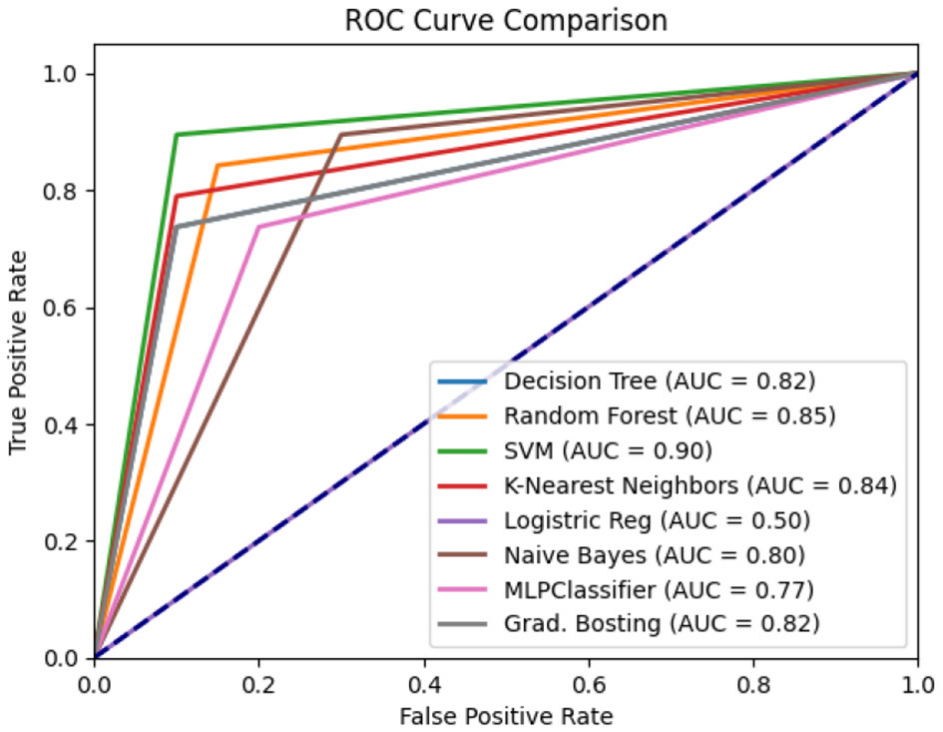
Experiment 4 ROC curve.

Experiment 4: In the final experiment, the prediction models were created using the same classifiers mentioned, and Xverse Voting Selector was applied to the WE feature. The total number of features used in this experiment was 16. The selected features are Q1, Q4, Q5, Q6, Q7, Q8, Q10, Q11, Q13, Q14, Q15, Q16, Q17, Q18, Q19, and Q20. The details of the questions are added to the Supplementary File S1. Of 16 features, 14 were similar to those selected in experiment 2. Conversely, Q17 and Q20 are the two new features that were not selected in experiment 2. The accuracy, recall, precision, and F1 of SVM reached 0.92 (95% CI: 0.88–0.95), 0.90 (95% CI: 0.86–0.98), 0.95 (95% CI: 0.86–0.99), and 0.92 (95% CI: 0.88–0.95), respectively, and has shown the best results, as can be seen in Table 11. Moreover, Figure 11 compares classifiers with selected features in the work environment using the Xverse Voting selector. The confusion matrix and ROC curves for the best-performing experiments have been included to validate the results further. Figure 12 shows the confusion matrix for experiment 4. The ROC curve of experiment 4 is shown in Figure 13. The highest ROC was achieved with SVM. LR has shown the lowest ROC value of 0.5.

**Table 11:** Models' performance using WE features (using Xverse voting selector).

**Xverse voting selector**				
**Classifier**	**Accuracy (95% CI)**	**Recall (95% CI)**	**Precision (95% CI)**	**F1 (95% CI)**
**With feature engineering (WE features)**				
DT	0.74 (0.72–0.83)	0.85 (0.73–0.92)	0.71 (0.67–0.82)	0.77 (0.72–0.85)
RF	0.87 (0.82–0.92)	0.85 (0.81–0.94)	0.89 (0.77–0.95)	0.87 (0.84–0.93)
LR	0.72 (0.59–0.83)	0.75 (0.56–0.92)	0.71 (0.63–0.88)	0.73 (0.59–0.84)
NB	0.82 (0.78–0.96)	0.75 (0.71–0.88)	0.88 (0.78–0.95)	0.81 (0.77–0.95)
KNN	0.87 (0.82–0.98)	0.89 (0.86–0.97)	0.84 (0.81–0.88)	0.86 (0.84–0.92)
MLP	0.79 (0.68–0.85)	0.85 (0.76–0.89)	0.77 (0.69–0.86)	0.81 (0.75–0.89)
SVM	0.92 (0.88–0.95)	0.90 (0.86–0.98)	0.95 (0.86–0.99)	0.92 (0.88–0.95)
GB	0.85 (0.80–0.91)	0.90 (0.79–0.98)	0.82 (0.77–0.97)	0.86 (0.81–0.91)

**Figure 14:**
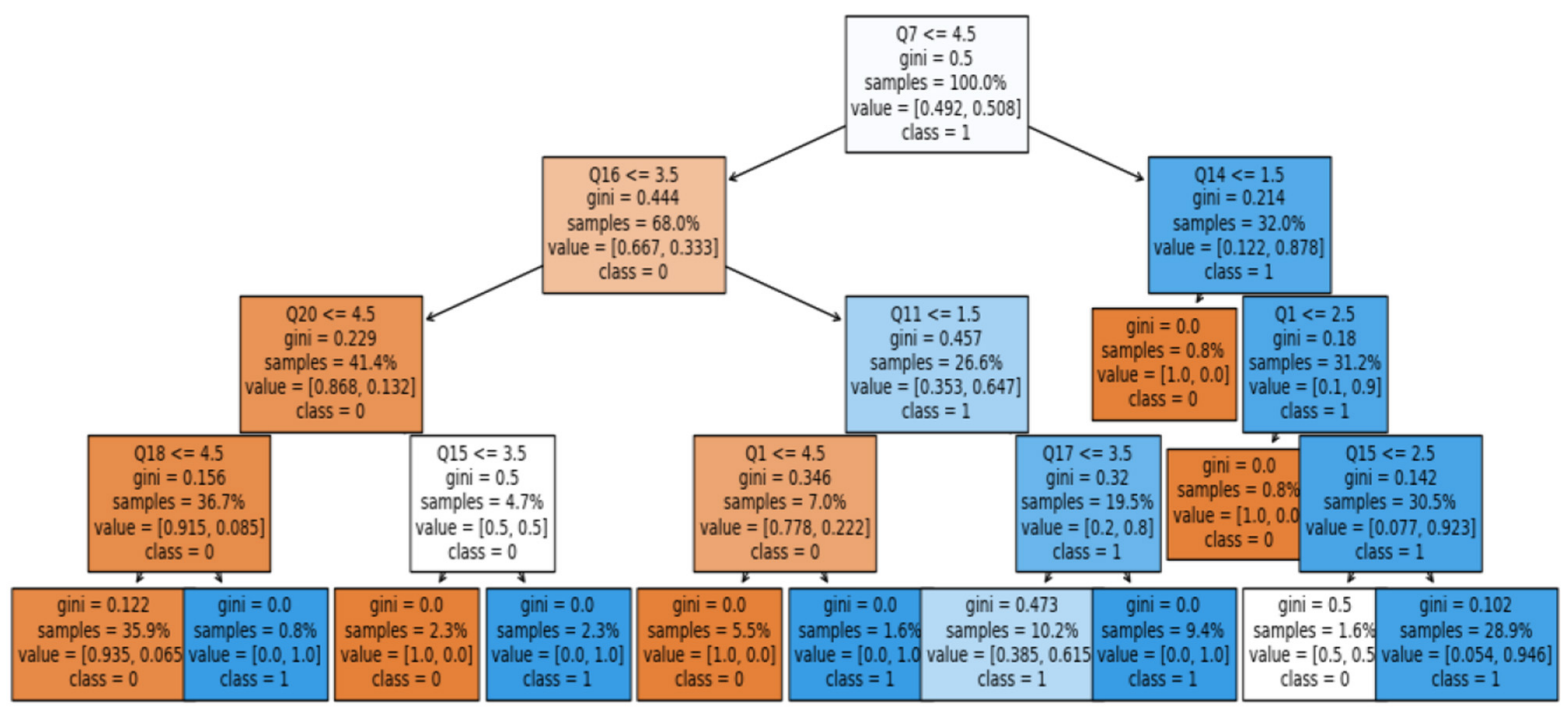
Extracted rules using the surrogate model plot method.

To summarize, we found that the WE-selected features could predict QoL among health-related employees. The SVM model produced the highest results in the current study. SVM is a black-box model; therefore, rules were extracted from it using a *post-hoc* explainable AI (XAI) approach. This approach adds transparency to the black-box model—the surrogate model rule-plot method used to extract the rules. Figure 14 shows the extracted rules using the surrogate model plot.

**Figure 15:**
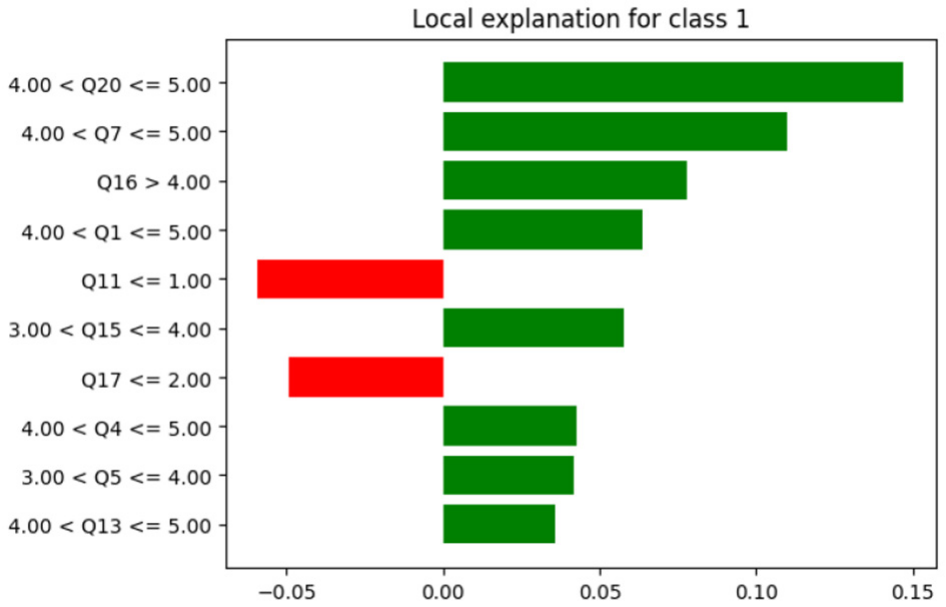
LIME for class 1.

Moreover, Local Interpretable Model-Agnostic Explanations (LIME) was used to illustrate why a particular decision was made. LIME provides the local explanation. Figure 15 presents the contribution to each attribute in the prediction for a specific record.

## 4 Discussion

In the current study, we aimed to develop an AI-based model to predict hospital employees' QoL using PWE features. Moreover, the study explored the impact of PWE features on prediction. The proposed model includes several PWE features that can predict HRQL level (Good/Bad) for healthcare practitioners. Several classifiers were used to develop the proposed model. SVM achieved the best results in experiments 3 and 4, where only PWE selected features using Xverse. The results in both experiments were identical with the accuracy [experiment 3 0.92 (95% CI: 0.89–0.99); experiment 4 0.92 (95% CI: 0.89–0.95)] and F1 [experiment 3 0.92 (95% CI: 0.89–0.99); experiment 4 0.92 (95% CI: 0.88–0.95)] but with different CI, whereas the recall and precision of 0.95 (95% CI: 0.92–0.1) and 0.90 (95% CI: 0.87–0.99) were achieved in experiment 3. However, in experiment 4, recall of 0.90 (95% CI: 0.86–0.98) and precision of 0.95 (95% CI: 0.86–0.99) were achieved.

In the literature, several studies have addressed the effect of the work environment on job satisfaction and job security using LR (41–44), with the highest reported accuracy of 0.86 for predicting job satisfaction (44). However, in our study, in addition to LR, several other ML classifiers were applied, and we found that the performance of other models was better (45). Compared to the LR, an improved, more accurate model was developed, achieving 1.0 precision with NB, indicating the model's ability to predict all cases correctly. In the current study, the NB classifier achieved the highest precision; however, it had relatively low accuracy and recall, at 0.81 (95% CI: 0.77–0.96) and 0.67 (95% CI: 0.58–0.96), respectively. Nevertheless, the SVM classifier provided more balanced results in terms of accuracy, recall, precision, F1, and ROC measurements [0.91 (95% CI: 0.87–0.97), 0.89 (95% CI: 0.93–0.97), 0.94 (95% CI: 0.79–0.95), 0.91 (95% CI: 0.86–0.97) and 0.90]. The model was developed using all PW and demographic features.

To enhance the prediction process, the ML approach uses feature engineering techniques to reduce the number of features (46). Mostly, feature engineering yields better results due to the use of only highly predictable features and the exclusion of the weak ones (47). Similar findings were made in the current study, where models developed with the selected features using the Xverse Voting Selector method produced the best results. SVM produced the highest precision [0.95 (95% CI: 0.86–0.99)] along with relatively high accuracy, recall, and F1 measurements [0.92 (95% CI: 0.88–0.95), 0.90 (95% CI: 0.86–0.98), and 0.92 (95% CI: 0.88–0.95)] with the Xverse voting feature selector and is considered the best model in the current study. Similarly, in (48), the SVM classifier produced the best results for predicting psychosocial risks among teaching employees. However, the target sample differed from that used in the current study.

The proposed study has successfully developed a high-accuracy model for predicting HRQL using only PWE factors. This coincides with the results of Canadian research, where they found that the organizational aspects, such as leadership style, organizational culture, and the way the healthcare provider communicates formally and informally, are more predictable features than personal or individual traits (42). Whereas in several other studies, the personal and demographic factors such as age, profession, duty hours, and years of experience have reduced the predictive ability (44, 49). The increased discrepancy in these factors could justify this, as the workplace and administrative settings are more alike among employees (43). Moreover, a study was conducted to identify workplace factors that predict nurses' quality and safety of patient care. RF with 10-fold cross-validation was used to analyze the 13 workplace factors. Among the significant factors were psychological protection, physical safety, and opportunities for engagement among the nurses. Like the current study, PW factors can precisely predict the QoL of the healthcare providers (50).

However, based on the statistical analysis in the study (29), the data collected from the cardiac unit were analyzed to determine the psychosocial environment. They found that to maintain good QoL, stakeholder collaboration is also required. Similarly, Sobhani et al. (30) found a relationship between the psychosocial environment and resilience levels among nurses.

Conversely, other studies found a strong relationship between nurses' work-life quality and their personal and individual attributes (44, 51). This implies that, regardless of attribute type, personal or work-related, they can be highly predictive, as shown in our feature engineering results, which included two personal information attributes: the employee's profession (which produced the highest information gain) and the employee's highest educational qualification. Correspondingly, six PWE-related attributes such as, whether the employee had contradictory demands placed on them at work, if the employee believes they work isolated from their colleagues, if the employee feels that they have the possibility of learning new things through their work, if the employee feels that their seniors discuss with them about how they will carry out their work, if the employee was worried about becoming unemployed, and finally if the employee has feedback regarding their work.

In a recent bibliometric analysis, it was found that nurses with QoL are less frequently investigated and require further study (4). This study advances research in this field by enabling healthcare organizations to predict, in advance, the status of their clinicians' QoL. This technique will allow for improvement before any misconduct results from poor QoL and will eventually improve healthcare services overall. As per the author's knowledge, Explainable AI (XAI) has not been investigated in any previous studies related to the current problem. Therefore, the current study is the pioneer in the application of XAI in predicting HQRL.

### 4.1 Limitations and future study

Nevertheless, the current study has produced an effective XAI-based prediction model; generalizability could be used with caution since the data were extracted from one study setting. In the future, external validation of the proposed model is needed to further assess its generalizability. An increased sample size across different study settings could improve the model's confidence. The proposed model can serve as a baseline for future studies in this domain. In the future, we aim to investigate the performance of the proposed model using multi-center and large datasets. Despite the predictive model's efficiency, it refrains from establishing any cause-and-effect relationships. In conclusion, the proposed model can be used to predict and improve healthcare providers' QoL, thereby advancing healthcare services.

Hospital administrators can use the proposed system to assess their employees' QoL. It can also help them identify the sectors that require improvement to enhance HRQL. This model empowers them to increase employee satisfaction, retention, and a healthy work environment.

## 5 Conclusion

A crucial indicator of overall health is the HRQL, which pertains to capturing the overall information on the physical and mental health of individuals, as well as the overall influence of health status on QoL. Measuring HRQL is essential, as it helps determine the burden of preventable diseases, disabilities, injuries, etc., which, in turn, helps monitor the progress of a nation's health objectives. A fundamental domain that significantly impacts one's HRQL is job-related factors, such as the PWE. A negative PWE severely compromises workers' HRQL. Moreover, if the work environment in which employees operate is toxic and uncollaborative, it directly affects workers' productivity and, in turn, impacts the company's profits. Therefore, PWE is one of the most influential factors that could directly affect the quality of services provided. Moreover, in healthcare, quality is a sensitive and highly dynamic topic. In the Saudi context, there is a lack of studies investigating the effects of PWE on workers' HRQL, specifically in the healthcare field. Therefore, this study examines the relationship between QoL, PWE, and healthcare quality in the Saudi context. To achieve this goal, a dataset was collected from a local hospital in Saudi Arabia, and ML models were deployed to predict the employee's QoL using PWE and the employee's demographic data. Hence, as far as the author is aware, this is the first study of its kind to predict HRQL based on a few elements of the PWE and some personal data using XAI. The proposed model has effectively predicted HRQL using the WE-selected features. During the experiments, it was found that work environment-related factors make the most significant contribution to HRQL. However, based on the information gained, the two personal attributes, i.e., profession and education, are also among the essential attributes.

Moreover, the surrogate model plot has explained the prediction. Thus, such a model is essential in any hospital to ensure employees' HRQL, which, in turn, will improve their productivity. This is especially important in health institutions, as employee productivity involves people's lives. It is worth noting that the findings of the current study were based on a dataset from a single hospital. Therefore, to further validate the current research's findings, we will evaluate the model's performance using multi-center data in the future. This will also increase the sample size. There is also a need to deploy the proposed model in a real-world environment to further validate the study's findings.

## Data Availability

The raw data supporting the conclusions of this article will be made available by the authors, without undue reservation.

## References

[1] Teles MAB Barbosa MR Vargas AMD Gomes VE de BL Martins AME Ferreira RC. Psychosocial work conditions and quality of life among primary health care employees: a cross sectional study. Health Qual Life Outcomes. (2014) 12:1–12. doi: 10.1186/1477-7525-12-72 24884707 PMC4122097

[2] Burton J World Health Organization. WHO Healthy Workplace Framework and Model: Background and Supporting Literature and Practices. Geneva, Switzerland: World Health Organization (2010).

[3] Lin WQ Wu J Yuan LX Zhang SC Jing MJ Zhang HS . Workplace violence and job performance among community healthcare workers in China: the mediator role of quality of life. Int J Environ Res Public Health. (2015) 12:14872–86. doi: 10.3390/ijerph121114872 26610538 PMC4661685

[4] Huang T Wu Y. A Bibliometric analysis of nurses' job satisfaction from 2004 to 2023. J Nurs Manag. (2025) 2025:4285361. doi: 10.1155/jonm/4285361 40337624 PMC12058320

[5] Anjum A Ming X Siddiqi AF Rasool SF. An empirical study analyzing job productivity in toxic workplace environments. Int J Environ Res Public Health. (2018) 15:1035. doi: 10.3390/ijerph15051035 29883424 PMC5982074

[6] Sturm H Rieger MA Martus P Ueding E Wagner A Holderried M . Do perceived working conditions and patient safety culture correlate with objective workload and patient outcomes: a cross-sectional explorative study from a German university hospital. PLoS ONE. (2019) 14:e0209487. doi: 10.1371/journal.pone.0209487 30608945 PMC6319813

[7] Abdi F Jahangiri M Kamalinia M Cousins R Mokarami H. Developing a model for predicting safety performance of nurses based on psychosocial safety climate and role of job demands and resources, job satisfaction, and emotional exhaustion as mediators. BMC Psychol. (2023) 11:1–13. doi: 10.1186/s40359-023-01223-1 37349826 PMC10288679

[8] Liau SY Shafie AA Ibrahim MI Hassali MA Othman AT Mohamed MH . Stages of change and health-related quality of life among employees of an institution. Health Expect. (2013) 16:199–210. doi: 10.1111/j.1369-7625.2011.00702.x 21645189 PMC5060655

[9] Hastings RP. Do challenging behaviors affect staff psychological well-being? Issues of causality and mechanism. Am J Mental Retard. (2002) 107:455–67. doi: 10.1352/0895-8017(2002)107<0455:DCBASP>2.0.CO;2 12323070

[10] Mathisen J Nguyen TL Jensen JH Rugulies R Rod NH. Reducing employee turnover in hospitals: estimating the effects of hypothetical improvements in the psychosocial work environment. Scand J Work Environ Health. (2021) 47:456–65. doi: 10.5271/sjweh.3969 34052852 PMC8504546

[11] Buder I Zick C Waitzman N. Health-related quality of life associated with physical activity: new estimates by gender and race and ethnicity. World Med Health Policy. (2016) 8:409–20. doi: 10.1002/wmh3.208

[12] Breevaart K Bakker AB. Daily job demands and employee work engagement: the role of daily transformational leadership behavior. J Occup Health Psychol. (2018) 23:338. doi: 10.1037/ocp0000082 28358569

[13] Daniels RJ. A generation at risk: young investigators and the future of the biomedical workforce. Proc Nat Acad Sci USA. (2015) 112:313–8. doi: 10.1073/pnas.1418761112 25561560 PMC4299207

[14] Scaffidi AK Berman JE. A positive postdoctoral experience is related to quality supervision and career mentoring, collaborations, networking and a nurturing research environment. High Educ. (2011) 62:685–98. doi: 10.1007/s10734-011-9407-1

[15] Chalofsky N Griffin MG. Work-Life Programs and Organizational Culture: The Essence of Workplace Community. Online Submission (2005).

[16] Sievers B. Motivation as a Surrogate for Meaning. Fachbereich Wirtschaftswiss, Berg Univ, Gesamthochsch (1984).

[17] Maslach C Jackson SE Leiter MP. The Maslach Burnout Inventory–Test Manual. Palo Alto, CA: Consulting Psychologists Press (1996).

[18] Kreitner R Kinicki A. Comportamento organizzativo. Milan, Italy: Apogeo editore (2004).

[19] Danish RQ Usman A. Impact of reward and recognition on job satisfaction and motivation: an empirical study from Pakistan. Int J Bus Manage. (2010) 5:159. doi: 10.5539/ijbm.v5n2p159

[20] Majernik ME Patrnchak JM. Rewards, recognition, and caregiver engagement at Cleveland Clinic. J Healthc Leadersh. (2014) 6:29–37. doi: 10.2147/JHL.S57063

[21] Altbach P. The costs and benefits of world-class universities. Int High Educ. (2003) 90:20. doi: 10.2307/40252583

[22] Ferrie JE Shipley MJ Stansfeld SA Marmot MG. Effects of chronic job insecurity and change in job security on self reported health, minor psychiatric morbidity, physiological measures, and health related behaviours in British civil servants: the Whitehall II study. J Epidemiol Community Health. (2002) 56:450–4. doi: 10.1136/jech.56.6.450 12011203 PMC1732160

[23] Cohen-Meitar R Carmeli A Waldman DA. Linking meaningfulness in the workplace to employee creativity: the intervening role of organizational identification and positive psychological experiences. Creat Res J. (2009) 21:361–75. doi: 10.1080/10400410902969910

[24] Gibbs Jr KD McGready J Griffin K. Career development among American biomedical postdocs. CBE—Life Sci Educ. (2015) 14:ar44. doi: 10.1187/cbe.15-03-0075 26582238 PMC4710405

[25] Lacerda SS Little SW Kozasa EH. A stress reduction program adapted for the work environment: a randomized controlled trial with a follow-up. Front Psychol. (2018) 9:668. doi: 10.3389/fpsyg.2018.00668 29867646 PMC5954607

[26] Bjorkman A Engstrom M Olsson A Wahlberg AC. Identified obstacles and prerequisites in telenurses' work environment–a modified Delphi study. BMC Health Serv Res. (2017) 17:1–11. doi: 10.1186/s12913-017-2296-y 28521743 PMC5437518

[27] Thorsen SV Madsen IEH Flyvholm M-A Hasle P. Associations between the workplace-effort in psychosocial risk management and the employee-rating of the psychosocial work environment–a multilevel study of 7565 employees in 1013 workplaces. Scand J Public Health. (2017) 45:463–7. doi: 10.1177/1403494817696377 28393650 PMC5495429

[28] Rezagholi M. Marginal socio-economic effects of an employer's efforts to improve the work environment. Ann Occup Environ Med. (2018) 30:1–7. doi: 10.1186/s40557-018-0212-5 29435336 PMC5793445

[29] Raycheva R Pavlova P Dimova R. Evaluation of psychosocial work environment among healthcare professionals in cardiac care units. Folia Med. (2024) 66:884–94. doi: 10.3897/folmed.66.e131663 39774366

[30] Sobhani S Tabanfar S Varmazyar S. Predicting resilience of hospital nurses based on workplace psychosocial factors. J Occup Health Epidemiol. (2024) 13:261–8. doi: 10.61186/johe.13.4.261

[31] Mendoza Bernal I Sánchez-Teruel D Robles-Bello MA Sarhani-Robles A Sarhani-Robles M. Predictors of resilience in healthcare workers during the COVID-19 pandemic: a longitudinal study comparing the first and second waves. BMC Psychol. (2023) 11:143. doi: 10.1186/s40359-023-01077-7 37131194 PMC10153033

[32] Ali AZ Alkubati SA Pasay-An E Alreshidi M Alrashidi N Alabonassir O . Investigating the level and predictors of nursing care quality and its correlation with workplace ostracism and innovative work behavior: approach for workplace and practical enhancement. BMC Nurs. (2025) 24:1–11. doi: 10.1186/s12912-025-03200-y 40375199 PMC12080122

[33] Alharbi A Alkubati SA Albaqawi H Ali AZ Hamed LA Mohammed S . Relationship between nursing work environment and clinical decision-making among Saudi nurses: psychological empowerment as mediator. BMC Nurs. (2025) 24:1–11. doi: 10.1186/s12912-025-03482-2 40598102 PMC12210569

[34] Holzinger A Kieseberg P Weippl E Tjoa AM. Current advances, trends and challenges of machine learning and knowledge extraction: from machine learning to explainable AI. Mach. Learn. Knowl. Extr. (2018) 11015:1–8. doi: 10.1007/978-3-319-99740-7_1

[35] Wu AW Rubin HR Mathews WC Ware JE Brysk Jr LT Hardy WD . A health status questionnaire using 30 items from the Medical Outcomes Study. Med Care. (1991) 29:786–98. doi: 10.1097/00005650-199108000-00011 1875745

[36] Pejtersen JH Kristensen TS Borg V Bjorner JB. The second version of the Copenhagen Psychosocial Questionnaire. Scand J Public Health. (2010) 38(suppl):8–24. doi: 10.1177/1403494809349858 21172767

[37] Rockwood TH Church JM Fleshman JW Kane RL Mavrantonis C Thorson AG . Fecal incontinence quality of life scale. Dis Colon Rectum. (2000) 43:9–16. doi: 10.1007/BF02237236 10813117

[38] Han J Kamber M Pei J. 9 - Classification: advanced methods. In:Han J Kamber M Pei J, editors. Data Mining, 3rd ed. The Morgan Kaufmann Series in Data Management Systems. Boston, MA: Morgan Kaufmann (2012). pp. 393–442. doi: 10.1016/B978-0-12-381479-1.00009-5

[39] Gorunescu F. Classification performance evaluation. Intell Syst Ref Libr. (2011) 12:319–30. doi: 10.1007/978-3-642-19721-5_6

[40] xverse·PyPI. Available online at: https://pypi.org/project/xverse/ (Accessed July 03, 2025).

[41] Arakawa C Kanoya Y Sato C. Factors contributing to medical errors among hospital nurses. Ind Health. (2011) 49:381–8. doi: 10.2486/indhealth.MS968 21372434

[42] Chamberlain SA Hoben M Squires JE Estabrooks CA. Individual and organizational predictors of health care aide job satisfaction in long term care. BMC Health Serv Res. (2016) 16:577. doi: 10.1186/s12913-016-1815-6 27737672 PMC5064796

[43] Faizin R Fitryasari R Wahyuni ED Nursalam N. Nurse's individual factors may predict quality of nursing work life (Qnwl) in clinical setting. Int J Psychosoc Rehabilitation. (2020) 24:9042–9. doi: 10.37200/IJPR/V24I7/PR2700894

[44] Kalisch B Tschannen D Lee H. Does missed nursing care predict job satisfaction? J Healthc Manage. (2011) 56:117–31. doi: 10.1097/00115514-201103000-00007 21495530

[45] Bozorgmehr A Thielmann A Weltermann B. Chronic stress in practice assistants: an analytic approach comparing four machine learning classifiers with a standard logistic regression model. PLoS ONE. (2021) 16:e0250842. doi: 10.1371/journal.pone.0250842 33945572 PMC8096078

[46] Anderson MR Antenucci D Bittorf V Burgess M Cafarella MJ Kumar . Brainwash: A Data System for Feature Engineering. CIDR (2013).

[47] Ozdemir S Susarla D. Feature Engineering Made Easy: Identify Unique Features from Your Dataset in Order to Build Powerful Machine Learning Systems. Packt Publishing Ltd. (2018). Available online at: https://books.google.com.sa/books?id=fiqSswEACAAJ

[48] Viloria A López JR Llinás NO Coronado LEL Mercado CV Sepulveda AMN . Prediction of psychosocial risks in teachers using data mining. In:Gunjan VK Senatore S Kumar A Gao X-Z Merugu S, editors. Advances in Cybernetics, Cognition, and Machine Learning for Communication Technologies. Cham, Switzerland: Springer (2020). pp. 501–8. doi: 10.1007/978-981-15-3125-5_50

[49] Berthelsen H Owen M Westerlund H. Does workplace social capital predict care quality through job satisfaction and stress at the clinic? A prospective study. BMC Public Health. (2021) 21:1320. doi: 10.1186/s12889-021-11320-8 34225680 PMC8259017

[50] Havaei F Ji XR Boamah SA. Workplace predictors of quality and safe patient care delivery among nurses using machine learning techniques. J Nurs Care Qual. (2022) 37:103–9. doi: 10.1097/NCQ.0000000000000600 34593739 PMC8860211

[51] Wallin AO Jakobsson U Edberg A-K. Job satisfaction and associated variables among nurse assistants working in residential care. Int Psychogeriatr. (2012) 24:1904–18. doi: 10.1017/S1041610212001159 22824091

