# Supplementary material for: Explainable machine learning for predicting hospital employees' quality of life using psychosocial work environment data
Source: Front Public Health. 2025 Dec 1;13:1529802. doi: 10.3389/fpubh.2025.1529802 (PMC12702951; doi:10.3389/fpubh.2025.1529802)
Supplement: Supplementary file 1 [file Table_1.docx]

**Work Environment related attributes.**

Q1: You have enough time for your work-task: (Quantitative Demands)

Q2: Your work require that you remember a lot of things: (Cognitive Demands)

Q3: Your work require that you have very clear and precise eyesight: (Cognitive Demands)

Q4: You have a large degree of influence regarding work performance: (Influence)

Q5: You decide when to take a break: (Recognition)

Q6: There is a good atmosphere between you and your colleagues: (Social community at work)

Q7: You get help and support from your colleagues: (Social support from colleagues)

Q8: Your superior talk with you about how will you carry out your work: (Role conflicts)

Q9: You work isolated from your colleagues: (Social community at work)

Q10: Your work emotionally demanding: (Emotional demands)

Q11: Your work require that you remember a lot of things: (Cognitive demands)

Q12: You have the possibility of learning new things through your work: (Possibilities for development)

Q13: You feel that the work you do is important: (Meaning of work)

Q14: You enjoy tilling others about your place of workplace: (Commitment to the workplace)

Q15: You informed well in advance about important decisions or changes related to your work-place: (Predictability)

Q16: You have say regarding your work: (Recognition)

Q17: Contradictory demands placed on you at work: (Role conflicts)

Q18: Your immediate superior is good at work planning: (Quality of leadership)

Q19: You worried about becoming unemployed: (Job insecurity)

Q20: How pleased are you with people you work with? (Job satisfaction) (Please choose one of the 5 options)

**Demographic information:**

1- Gender

2- Age:

3-Profession:

4- Your highest educational qualification:

5- Shift:

6- Experience

7- Nationality
